# A study on the clusterability of latent representations in image pipelines

**DOI:** 10.3389/fninf.2023.1074653

**Published:** 2023-02-16

**Authors:** Adrian Wheeldon, Alexander Serb

**Affiliations:** Centre for Electronics Frontiers, School of Engineering, University of Edinburgh, Edinburgh, United Kingdom

**Keywords:** machine learning, symbolics, clustering, convolutional neural networks, autoencoders, artificial intelligence, cognitive computing

## Abstract

Latent representations are a necessary component of cognitive artificial intelligence (AI) systems. Here, we investigate the performance of various sequential clustering algorithms on latent representations generated by autoencoder and convolutional neural network (CNN) models. We also introduce a new algorithm, called Collage, which brings views and concepts into sequential clustering to bridge the gap with cognitive AI. The algorithm is designed to reduce memory requirements, numbers of operations (which translate into hardware clock cycles) and thus improve energy, speed and area performance of an accelerator for running said algorithm. Results show that plain autoencoders produce latent representations which have large inter-cluster overlaps. CNNs are shown to solve this problem, however introduce their own problems in the context of generalized cognitive pipelines.

## 1. Introduction

Sensory pipelines can be broadly understood as signal processing cascades that receive raw sensor data (e.g. pixel intensities of an image) and refine it until a symbolic representation emerges. Symbolic representations are generally understood as representations that contain high amounts of semantic information and are typically represented as hypervectors (Kanerva, 1988; Ge and Parhi, 2020; Neubert et al., 2021). A typical way of implementing a pipeline is by use of artificial neural networks (ANNs). These span from the typical convolutional neural networks (CNNs) that classify input images (Lecun et al., 1998), to more elaborate systems that, for example, attempt to extract partial information such as color and shape separately from individual objects (Frady et al., 2020), and arguably even to autoencoders that map input data to a latent space that ideally supports segmentation into areas of reasonably well-defined semantics (Newson et al., 2020).

**Figure 1:**
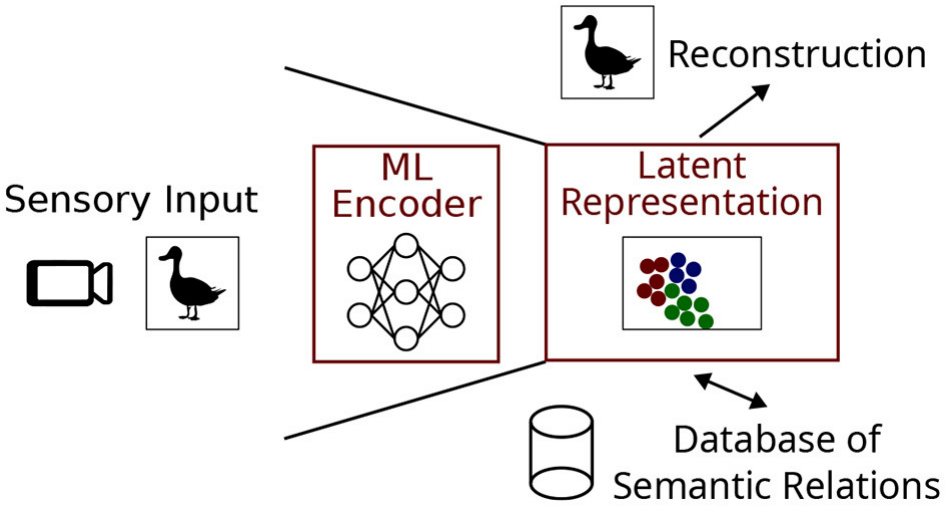
A sensory pipeline based on image input useful for cognitive artificial intelligence. The machine learning (ML) encoder and latent representation are the focus of this work.

In this paper we focus on latent representations and the process of generating them through a combination of ANN and clustering algorithms. Our basic set-up is as follows: Sensory input in the form of an image is used to create the vision pipeline shown in Figure 1, although pipelines can process any type of input including audio or text. The job of the encoder is to compress the sensory input into a latent representation which can be stored in a database of semantic relations. Another desired aspect of the system is the ability to reconstruct an approximation of the original sensory input using the latent representation, as it would form a convenient way of allowing the system to ‘imagine' known objects in the absence of direct visual stimulation and potentially transform them (e.g. mental rotation).

We apply cluster algorithms to the latent representations emerging from autoencoder action to cast a net around the learned examples from each class of the dataset. These nets are expected to encompass new datapoints belonging to their corresponding classes, including new variants such as in pose, illumination and orientation, allowing the system to capture them during inference. This is the principle of preserving equivariance of the sensory input through the encoding process (Cohen and Welling, 2017), and is necessary for the clustering process to effectively compress the input whilst maintaining intra-class similarity and inter-class dissimilarity.

Many clustering algorithms make multiple passes over the data (Imani et al., 2019), requiring some or all of the data to be stored for later access. However, it is not practical to implement such schemes in hardware striving for minimum energy, since data storage demands high amounts of energy. Therefore, we are most interested in single-pass sequential clustering algorithms where data can be clustered *online* as it arrives. The Leaders algorithm is a single-pass, sequential, distance-based clustering algorithm which was introduced by Hartigan (1975, p. 74). In brief, the algorithm functions as follows: 1) if the new datapoint lies within radius *r* of a cluster, add the datapoints to that cluster, else create a new cluster with that datapoint as the center; 2) repeat 1) until all datapoints are allocated to clusters. Modifications to this algorithm include BSAS, MBSAS and TTSAS (basic, modified and two-threshold sequential algorithmic schemes) (Theodoridis and Koutroumbas, 2006), which respectively introduce a new parameter, *maximum number of clusters*; a second pass of the dataset; and a second threshold. These will be discussed in detail in Section 1.1.

On the back of these clustering algorithms, we present a new algorithm, called *Collage*. The aims of Collage are to produce a clustering algorithm with the following abilities:

1. Process an unknown number of classes.
2. Process datapoints in a sequential, *online* manner.
3. Process datapoints without supervision or *a priori* information.
4. Can be implemented in a hardware-friendly manner.
5. Has a low number of parameters to optimize.

Collage makes improvements over the aforementioned sequential clustering algorithms in hardware friendliness. The competitive algorithms perform averaging of points upon every allocation of a new cluster, which is an operation that takes a significant portion of system energy to perform. Additionally, the multi-pass approach of MBSAS and TTSAS adds a multiplier to the memory size requirements, as deferred datapoints must be stored until they are eventually processed. Collage solves this problem by passing over the data only once as it arrives. Table 1 summarizes the features of the sequential clustering algorithms of interest. The main focus of the algorithm, however, is the introduction of new functionality to enable semantic linking of clusters. This is discussed in more detail in Section 2.

**Table 1:** Comparison of online clustering algorithms.

	**New center strategy**	**Parameters**	**Dataset passes**
Leaders^*a*^	First outside r	r	1
**Collage**	First outside green radius	Green radius, Amber radius	1
BSAS^*b*^	First outside r	r, max_clusters	1
MBSAS^*b*^	First outside r	r, max_clusters	2
TTSAS^*b*^	First outside r2	r1, r2	2+

^*a*^Hartigan (1975). ^*b*^Theodoridis and Koutroumbas (2006).

In this work, we investigate the quality of the latent representations generated by different machine learning models; namely the autoencoder, and the CNN; for use in the vision pipeline system. We do this by measuring the homogeneity of the clusterings generated by the various models and clustering algorithms; as well as analyzing the cluster sizes, diameter distributions, and the total usage of the latent space. We also investigate the effects of data augmentation on the autoencoder model and its clusterings. We use the k-Means clustering algorithm as a baseline for our experiments. Although it does not fit our criteria of a single-pass and sequential algorithm, it nevertheless provides a useful upper bound for clustering quality in our experiments.

### 1.1. BSAS, MBSAS, and TTSAS algorithms

BSAS, MBSAS, and TTSAS are distance-based clustering algorithms introduced by Theodoridis and Koutroumbas (2006) and extend the Leaders algorithm (Hartigan, 1975 p. 74) introduced previously. New clusters are defined using points which lie outside the threshold of existing cluster centers. A cluster center is updated when a new datapoint is added to that cluster. To generate the new cluster center, the current center is averaged with the new datapoint. The latter is an important distinction from Collage algorithm.

The BSAS and MBSAS algorithms allow a maximum number of clusters to be specified. Once the maximum number of clusters has been reached, no new clusters are created and datapoints can only be added to existing clusters. To make proper use of this parameter, it is necessary to have a priori information about the dataset. Since one of the goals of our algorithm is to be agnostic to the number of clusters, in our experiments we set this parameter equal to the number of datapoints in the dataset. This has the affect that the algorithm can create as many clusters as deemed necessary.

In addition to the above, the MBSAS algorithm takes two passes of the data. On the first pass, all of the cluster centers are initialized. This is done such that every datapoint which is farther than the threshold from the existing cluster centers creates its own cluster center. Datapoints which lie within the threshold of a cluster center are skipped during the first pass. On the second pass, all skipped datapoints are processed and added to their nearest clusters.

The TTSAS algorithm goes further by utilizing two radii and taking several passes of the dataset. Datapoints which lie between the two radii are deemed to be in a “gray” area, and their assignment to a cluster is deferred to the next algorithm iteration. If such a datapoint will later lie within the first radius of a cluster, it shall become part of that cluster, otherwise it will eventually make a cluster of its own. Datapoints which lie outside the outermost radius form a new cluster immediately.

## 2. The collage algorithm

Collage is a sequential clustering algorithm controlled by two parameters: green radius and amber radius. It operates on data in an online manner and datapoints are processed as they are received or generated upstream. The algorithm is shown in Algorithm 1 and is summarized by the following: The first datapoint forms the first cluster and becomes its center. As new datapoints arrive, their distance from the existing cluster centers is measured. If the datapoints lie within a certain radius of an existing cluster, defined as the *green radius*, it is added to that cluster. Should multiple cluster centers compete (a datapoint is within the green radius of multiple cluster centers), the nearest neighbor is chosen. Furthermore, if there are equidistant cluster centers, the earliest-defined cluster takes precedence. If the datapoint lies outside the green radius of all clusters, the datapoint forms a new cluster and becomes its center. If the datapoint is not contained within the amber radius of any other cluster, then no further action is taken, and the algorithm behaves like BSAS.

**Algorithm 1:** Collage.

centers ← {data[0]}
for all *x*∈ data **do**
*n*← nearest neighbor of *x*∈ centers ⊳ Earliest-defined center if equidistant
*d*← euclidean distance *x*, *n*
if *d* < green radius **then**
Append *x* to cluster containing *n*
else
Append *x* to centers ⊳ Create new cluster
end **if**
for all *M*∈ centers where (distance *x* to any center < amber radius) **do**
Semantically connect *M*
end **for**
end **for**

**Figure 2:**
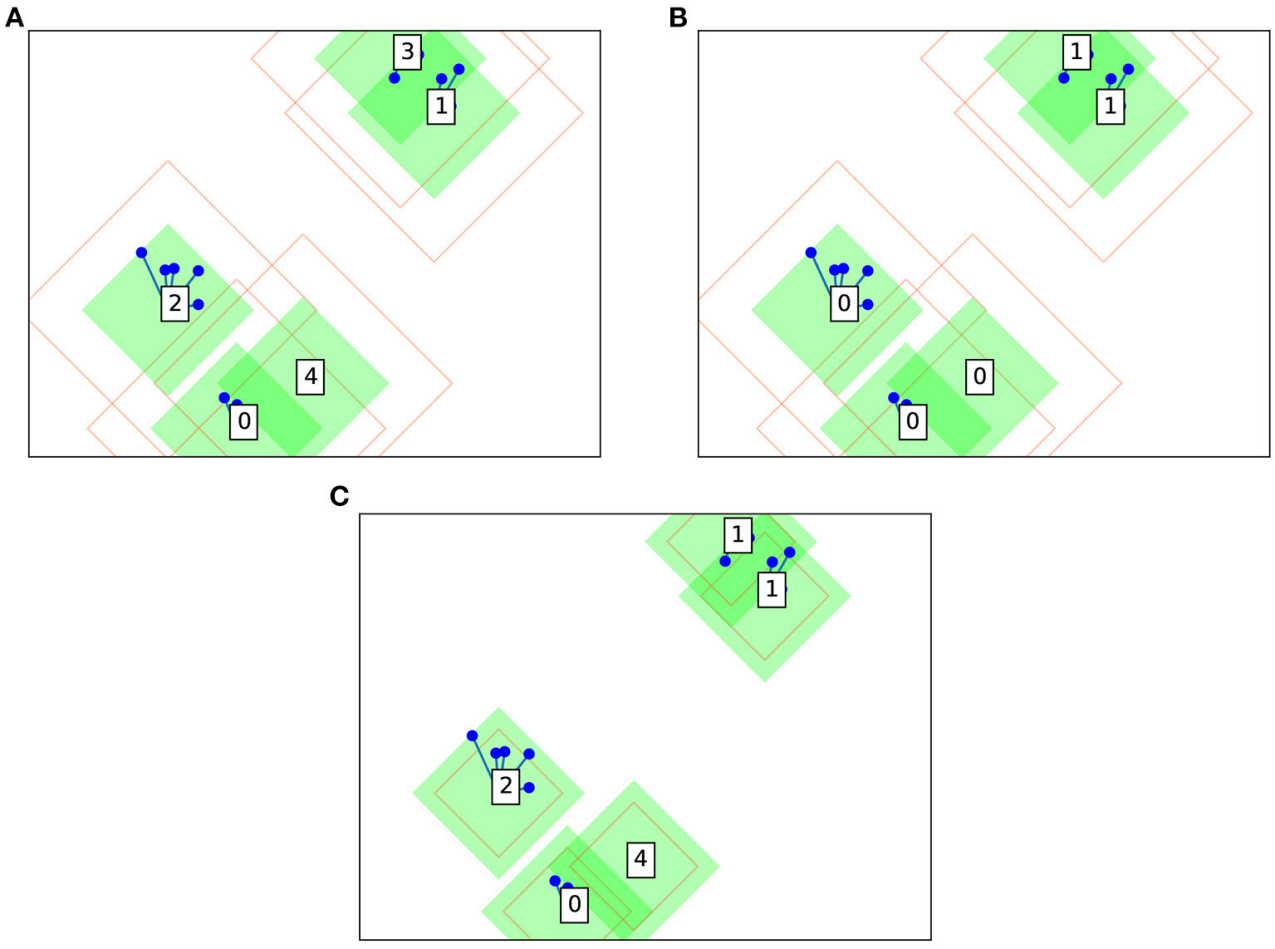
Example Collage clustering in two dimensions on the same dummy dataset: **(A)** without semantic merging; **(B)** with semantic merging; and **(C)** with semantic merging and smaller amber radius.

Furthermore, in the case where a datapoint lies within the amber radius of two or more clusters, these clusters are deemed to be semantically connected—that is, the clusters remain conceptually distinct, but share a common meaning. Each cluster represents a “view” of a contiguous visual object. Beyond the algorithm and as part of the artificial intelligence (AI) pipeline, these views can be semantically connected in a backend database of relations to define the visual objects. As far as the clustering algorithm is concerned the clusters remain distinct—the idea being that slight transformations of an object, such as rotation or illumination change, would be captured by this mechanism. In purely mathematical terms (i.e. ignoring the mechanic of the backend database), the semantic connection manifests as a merging of the clusters. Contrast Figure 2A without merging; and Figure 2B with semantic merging of clusters. An amber radius of zero means that semantic merging is deactivated. In this case, Collage algorithm will produce clusterings similar to those of Leaders algorithm. The behavior of the algorithm based on the location of the arriving datapoint is summarized in Table 2. The choice of green and amber radii depends on the distribution of the dataset, and trades off accuracy with number of clusters generated. The larger the values for these radii, the fewer clusters will be generated, as each cluster will tend to accumulate more datapoints as it covers a larger portion of the latent space.

**Table 2:** Behavior of collage algorithm when a new datapoint arrives.

**Condition**	**Outcome**
*d* < green ≤ amber	Datapoint assigned to cluster.
green ≤ *d* < amber	Datapoint creates new cluster and initiates semantic merge.
green ≤ amber ≤ *d*	Datapoint creates new cluster.

An example clustering is shown in Figure 2A using a dummy dataset based on random blobs. Cluster centers are shown as boxes with a numerical label along with their surrounding green and amber radii. Note that these radii take the shape of a diamond, since Collage is designed to primarily use L1 geometry (also know as Manhattan or city block geometry). Hardware implementation favors L1 geometry over L2 due to avoidance of power and area intensive mathematical operations, i.e. square and square root, when calculating distances between points. Other datapoints are shown as blue circles and lines connect the datapoints to their associated cluster centers.

The amber radius is permitted to be less than the green radius as illustrated in Figure 2C. In this scenario, and when a datapoint lies between green and amber radii of its cluster, the normal rules apply. That is, the datapoint becomes part of the cluster however it cannot initiate a semantic merge with another cluster, as it is not within the amber radius.

Other generalizations of the algorithm are possible for supporting cases where the amber radius is smaller than the green radius. One such generalization would have points lying between the amber and green radii both automatically labeled as prescribed by the center of the existing nearest-neighbor class and generating a new cluster center (expanding the cluster). Another generalization would simply have points between amber and green create new clusters, competing with the original center of the radii.

## 3. Experimental setup

We test the clustering algorithms firstly on the raw MNIST dataset to obtain baseline performance. The raw dataset comprises 60,000 training images of 28 x 28 grayscale pixels. For clustering, this is flattened to a single vector of 784 pixels and normalized to the range [0, 1] in floating point. We then test the algorithms on latent representations of the dataset generated by: 1) an autoencoder; and 2) a CNN.

**Figure 3:**
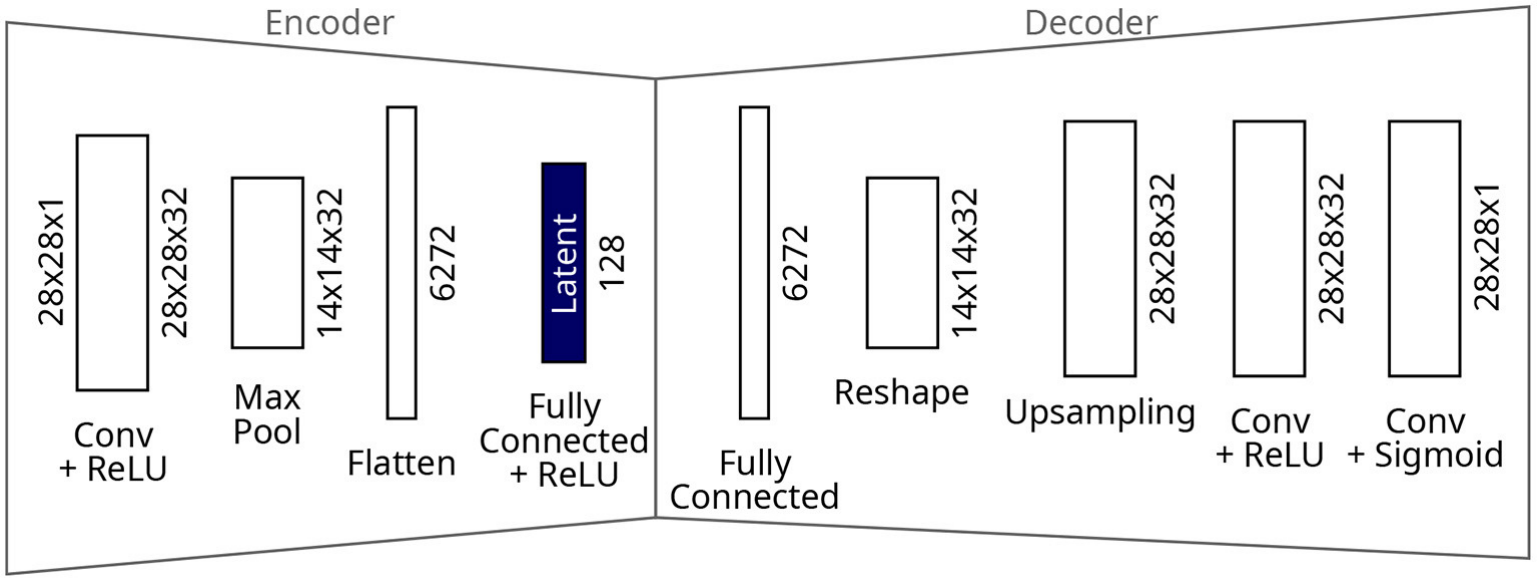
Architecture of the autoencoder for the MNIST dataset.

The autoencoder model (Figure 3) consists of convolutional layers which form the encoder; a bottleneck from which the latent representations are extracted, which is narrower than the input width; and deconvolutional layers which form the decoder. The aim of the autoencoder is to reconstruct the input exactly, using a pixel-by-pixel mean-squared error loss, whilst the amount of information is reduced through the bottleneck. The autoencoder model used on the MNIST experiments consists of one convolutional layer with 32 filters and rectified linear unit (ReLU) activations, followed by a max pooling layer. The resulting 14 x 14 x 32 tensor is flattened before the fully-connected layer of 128 neurons which forms the bottleneck. ReLU activations which saturate at 1.0 complete the latent layer and encoder. For the decoder, the 6,272-neuron bottleneck is reshaped back to 14 x 14 x 32 before upsampling and a convolutional layer of 32 filters. The decoder is completed by a further convolutional layer of one filter to restore the original data dimensions of 28 x 28 x 1, and finished with sigmoid activations to constrain the outputs in the range [0.0, 1.0]. The autoencoder was trained for 100 epochs resulting in a final training loss of 1.1 × 10-3 on the MNIST dataset.

**Figure 4:**
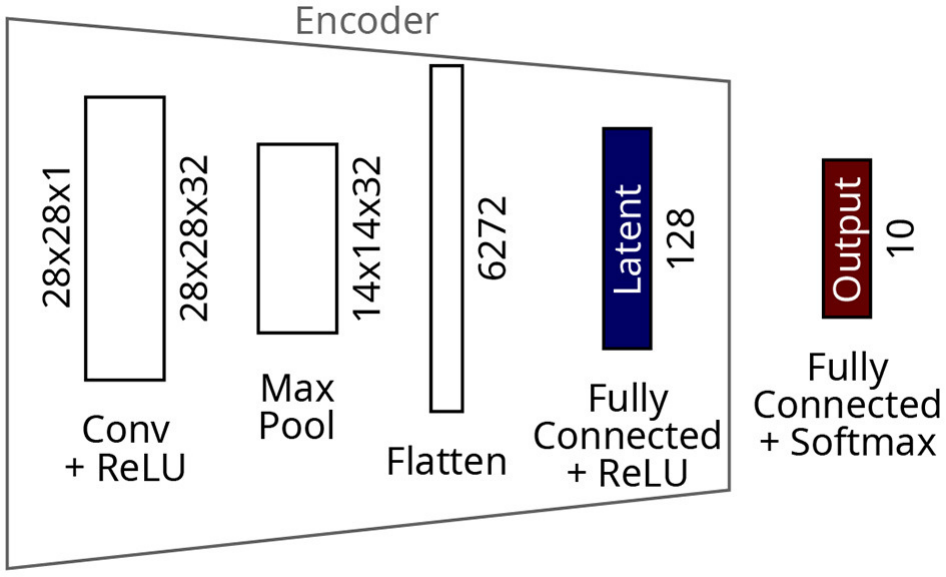
Architecture of the CNN for the MNIST dataset.

The CNN model (Figure 4) comprises one convolutional layer with 32 filters and ReLU activations, followed by a max pooling layer. The resulting 14x14x32 tensor is flattened before a dense layer of 128 neurons. ReLU activations which saturate at 1.0 complete the latent layer and the encoder. A dense layer with ten neurons and softmax activations makes the output layer. The CNN was trained for 100 epochs resulting in a final training loss of 3.7 × 10-8 on the MNIST dataset.

The encoder layers and the bottleneck are kept the same between both autoencoder and CNN models to allow direct comparison of results. The number of latent neurons is chosen based on the dataset and model. There must be sufficient latent neurons to preserve enough information from the input to enable classification and reconstruction, whilst providing a reasonable level of compression. In this example we use 128 latent neurons which gives low training losses as previously presented, and image reconstructions which are perceptibly very close to the originals.

In the case of the sequential clustering algorithms, data ordering is important since each datapoint is processed in order of arrival. To take into account the effects of data ordering, we run the experiments on a ten-fold cross validation, logging the mean and standard deviation of the metrics such as homogeneity and number of clusters. The k-Means experiments are run once since data ordering has a negligible effect on the outcome.

### 3.1. Data augmentation

**Figure 5:**
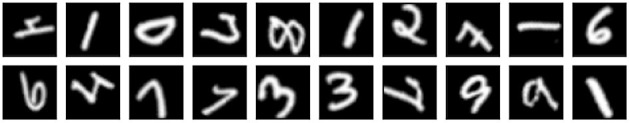
Examples from our augmented MNIST training set.

For one select experiment, we train the neural network (NN) models using an augmented version of the MNIST dataset. Each sample from the original dataset is repeated ten times, each time with a different, random combination of transformations applied. The transformations include a translation in both *x* and *y* planes up to three pixels in either direction; and a rotation around the center of up to ±π/2 radians. New pixels that must be introduced into the transformed image are filled with the value 0.0. For the augmentation experiments, the validation dataset remains untouched from the original. Examples from the augmented dataset are shown in Figure 5.

### 3.2. Cluster algorithm parameter optimization

The k-Means algorithm has only one parameter, which is the number of clusters to which the data is fit. In contrast, Collage does not produce a set number of clusters since it is designed to adapt online to the data. It takes two parameters which are the radii used to define the green and amber regions of each cluster. The parameters of the algorithms are summarized in Table 3. The BSAS and MBSAS algorithms require a maximum number of clusters to be given. As previously discussed, our clustering aim is to be able to handle an unknown number of clusters, therefore in our experiments this parameter is set to the number of datapoints in the dataset. This effectively disables the mechanism of limiting the number of clusters in the algorithm to simulate an algorithm which is not dependent on *a priori* dataset information. For deployment, this mechanism should be disabled internally so that the *maximum clusters* parameter need not be supplied for these algorithms.

**Table 3:** Summary of clustering algorithm parameters.

**Algorithm**	**Parameter**	**Description**
k-Means	Number of Clusters	Fixed number of clusters to generate.
Leaders	r	Points within are absorbed.
Collage	Green Radius	Points within are absorbed.
	Amber Radius	Points within can cause cluster merges.
BSAS, MBSAS	r	Points within are absorbed.
	max_clusters	New clusters cannot be formed when limit reached.
TTSAS	r1	Points between r1 and r2 defer to next iteration.
	r2	Points outside r2 form a new cluster.

### 3.3. Clustering analysis

The main metric we use to measure the clustering performance is homogeneity. Homogeneity is satisfied (homogeneity score of 1.0) if all clusters contain only datapoints which are members of a single ground-truth class. The lower bound for homogeneity is zero, occurring when all cluster members belong to different ground-truth classes. Cluster sizes are measured by diameter, which is calculated by measuring the largest distance between two datapoints in the same cluster. To analyse the distributions of cluster sizes and samples within a cluster, we chose the parameters for Collage before matching the number of clusters for k-Means.

## 4. Results

**Figure 6:**
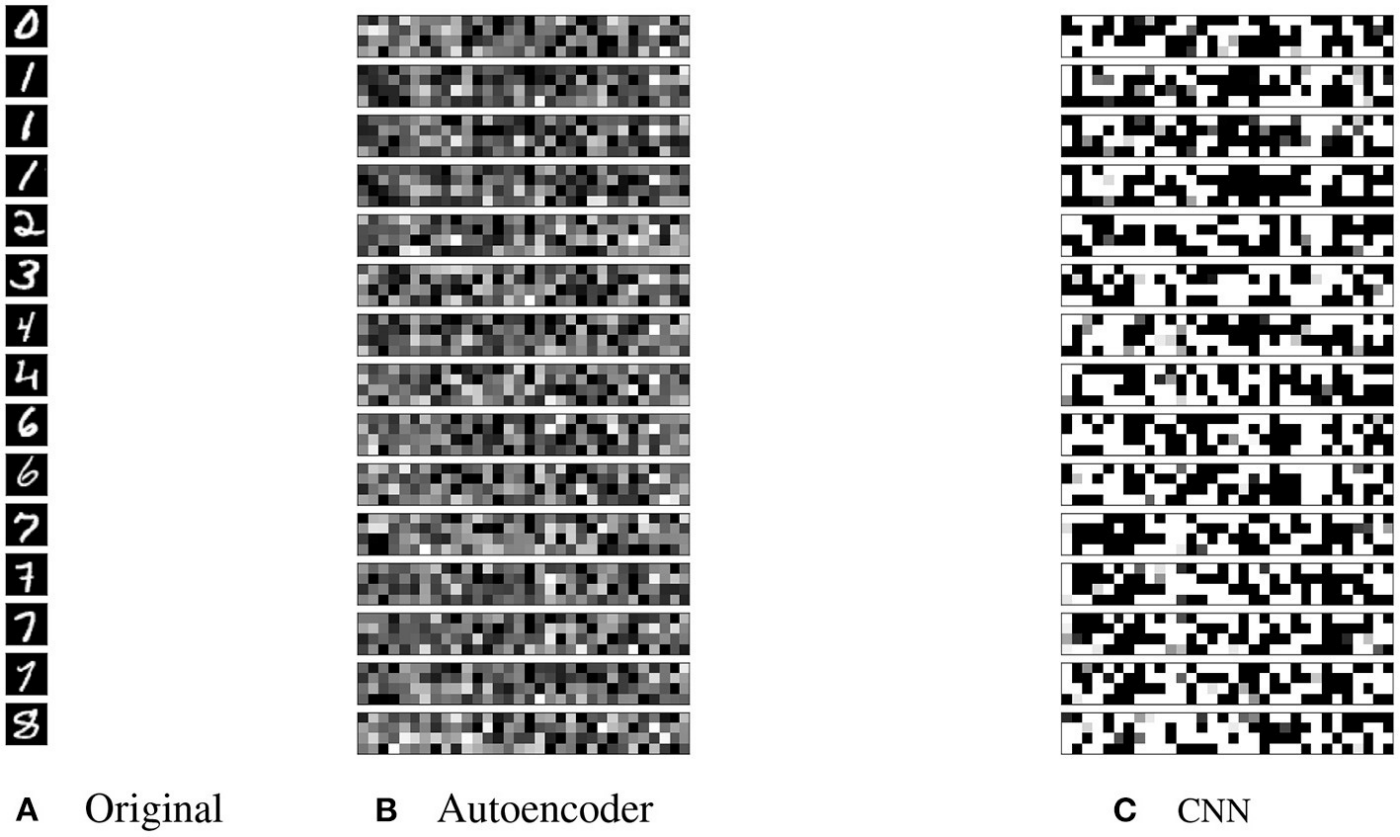
**(A)** Selection of the original dataset; **(B)** the latent representations generated by the autoencoder; and **(C)** the latent representations generated by the CNN. Black is value 0.0, white is value 1.0.

A selection of the latent representations generated by our models for the MNIST dataset are shown in Figure 6. The latent representations from the autoencoder (Figure 6B) are distributed more evenly throughout the range of possible values [0.0, 1.0] compared with the CNN. An average of only 16/128 latent neurons saturate in the autoencoder model on the same dataset. For representations generated by the CNN (Figure 6C), some visual intra-class similarities can be seen. An average of 119/128 of the values saturate at 0.0 or 1.0 when tested on the MNIST dataset with and without augmentation. This suggests that the model is not making full use of the latent space that is available. This is also evident in the clustering of the CNN latent space that we will discuss later.

### 4.1. Raw dataset clustering

Before exploring different NN architectures for producing latent representations, we test the performance of the clustering algorithms on the raw datasets. This gives a baseline that we can use to determine how effective the architectures are at compressing the examples into the latent space.

**Figure 7:**
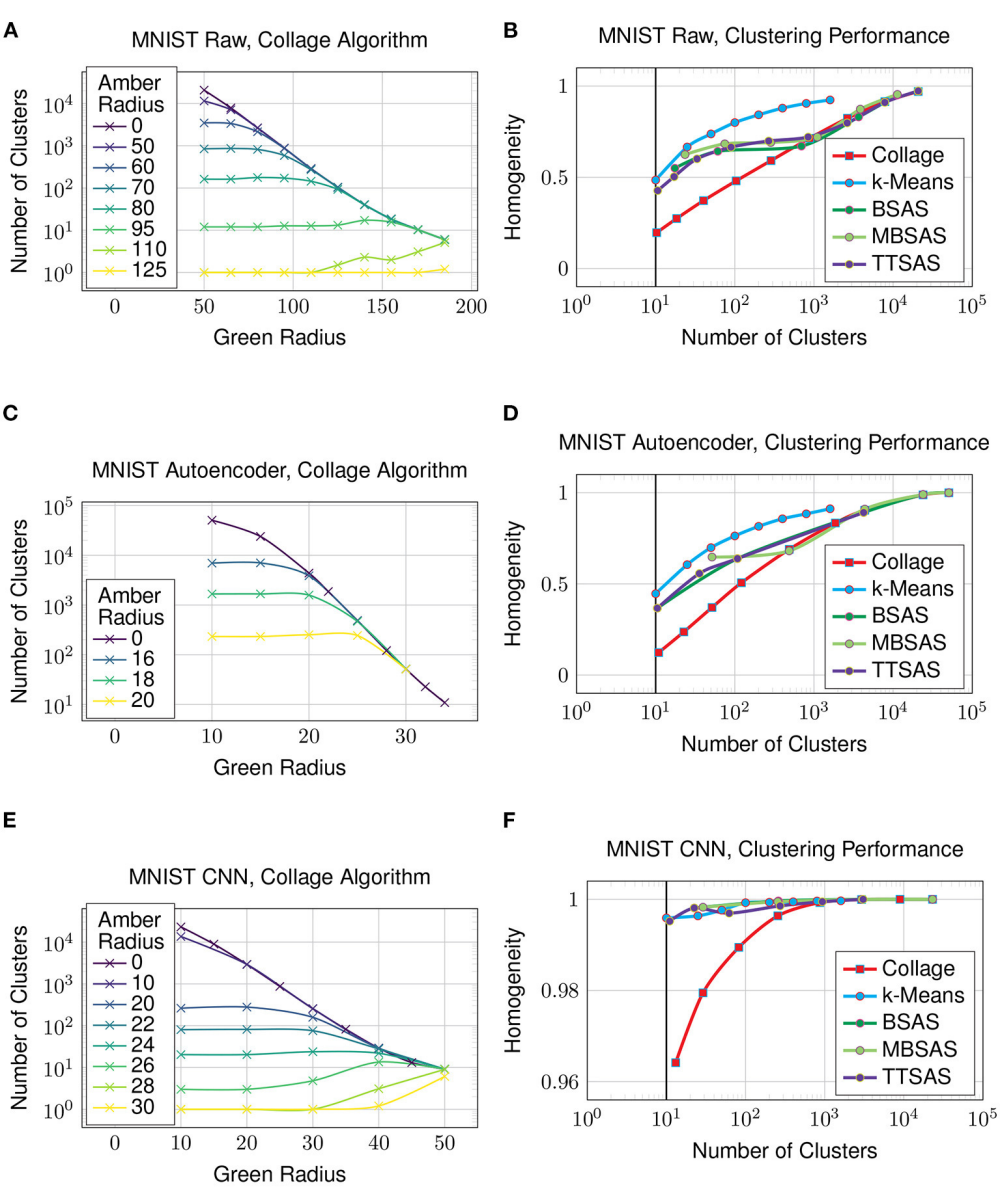
Clustering on the MNIST dataset. Solid vertical line in **(B, D, F)** denotes the case where clusters obtained is equal to number of ground truth classes. **(A)** MNIST raw, collage algorithm, **(B)** MNIST raw, clustering performance, **(C)** MNIST autoencoder, collage algorithm, **(D)** MNIST autoencoder, clustering performance, **(E)** MNIST CNN, collage algorithm, and **(F)** MNIST CNN, clustering performance.

Starting with the MNIST dataset, Figure 7A shows how the green and amber radii of the Collage algorithm affect the number of clusters generated. As all algorithms are sensitive to dataset ordering, with the exception of k-Means, the plot shows the mean number of clusters over ten different dataset orderings. The uppermost line shows Collage without amber merges—this produces similar clusterings to the Leaders algorithm. Each line beneath represents a different value for the amber radius. Since merged clusters are left otherwise unchanged, the homogeneity cannot be improved with increasing amber radius.

The trend between the number of clusters generated by an algorithm and the homogeneity of its clustering is seen in Figure 7B. k-Means sets the performance baseline for this experiment, as it has the advantage of analyzing the whole dataset before settling on a clustering. We do not expect any of the sequential, few pass algorithms to exceed k-Means' performance. Again, as the sequential algorithms are sensitive to dataset ordering, the mean homogeneity is taken over ten different dataset orderings. TTSAS is more robust to data ordering variations due to its additional radius, and this will be explored in Section 4.5, however no homogeneity advantage is gained over BSAS or MBSAS in the average case as seen in the plots. In the case that the number of generated clusters equals the number of classes in the dataset, even the best clustering achieves only 0.5 homogeneity. This poor clustering is not unexpected, given the raw dataset comprises an array of pixels whose values have a large amount of overlap.

### 4.2. Autoencoder

Now we investigate how the clustering algorithms perform on a dataset which is encoded to a latent representation. In this experiment, the latent representation is obtained by extracting the outputs from the bottleneck of the autoencoder from Figure 3. Figure 7C shows the number of clusters generated by Collage with varying green and amber radii—many more clusters are generated compared to what is achievable on the raw dataset. The clustering performance, shown in Figure 7D, is *worsened* for all algorithms in comparison to the raw dataset. The data has been compressed through the bottleneck, however the result is many overlapping clusters in the latent space, and thus decreased cluster homogeneity.

### 4.3. CNN

In this experiment, the latent representation is obtained from the penultimate layer of the CNN from Figure 4. Figure 7E shows that the number of clusters generated by Collage is now more on par with those from the raw dataset. A reasonable number of clusters, close to the number of ground truth classes, is now achievable with much reduced green and amber radii. Clustering performance on the latent representation in Figure 7F is vastly improved over both the raw dataset and the latent representation generated using an autoencoder. Homogeneity is 0.996 for the k-Means algorithm where the number of clusters equals the number of classes in the dataset. The other sequential algorithms also perform very well on this representation. The performance of Collage begins to drop off below 100 clusters, however homogeneity is maintained above 0.95 for all number of clusters exceeding the number of classes in the dataset. The results show that the CNN is maintaining separation between clusters and thus preventing cluster overlaps.

### 4.4. CIFAR10 dataset

**Figure 8:**
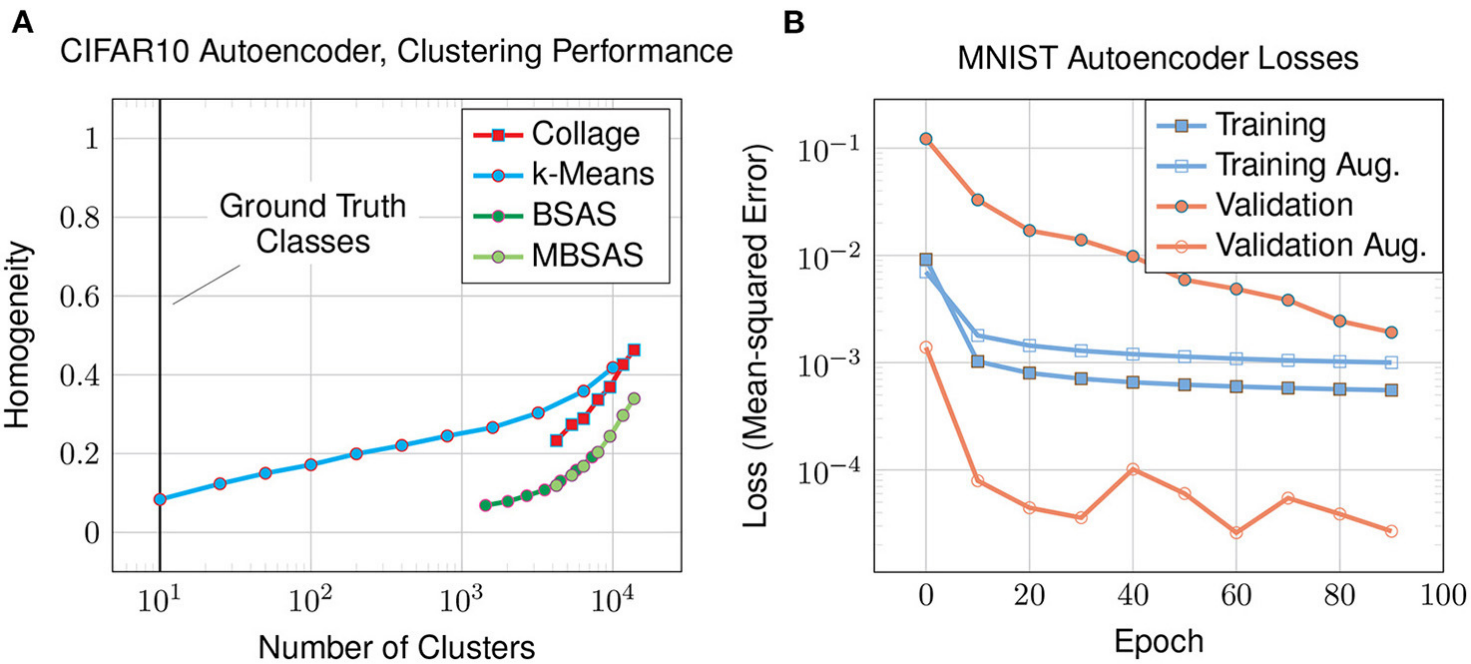
The latent-encoded CIFAR10 dataset using an autoencoder: **(A)** homogeneity of clusters compared to the number of clusters; and **(B)** training and validation losses for augmented (aug.) and non-augmented datasets.

In this experiment we look at the clustering performance on the CIFAR10 dataset to see how the autoencoder and Collage algorithm performs on a more demanding dataset with higher input dimensionality. The dataset comprises 60,000 full color images of dimensions 32 x 32 x 3 that are arranged into ten classes of animals and vehicles with backgrounds. For this reason, we modify the autoencoder architecture used in previous experiments (Figure 3) by adding two additional convolutional and deconvolutional layers, resulting in a total of three. The fully-connected layer preceding the bottleneck now consists of 8,192 neurons. We increase the number of latent neurons to 1,024 to achieve a reconstruction loss of 3.4 × 10-4 after 100 epochs. The plot of homogeneity against number of clusters generated (Figure 8A) shows poor homogeneity.

### 4.5. Cluster analysis

**Figure 9:**
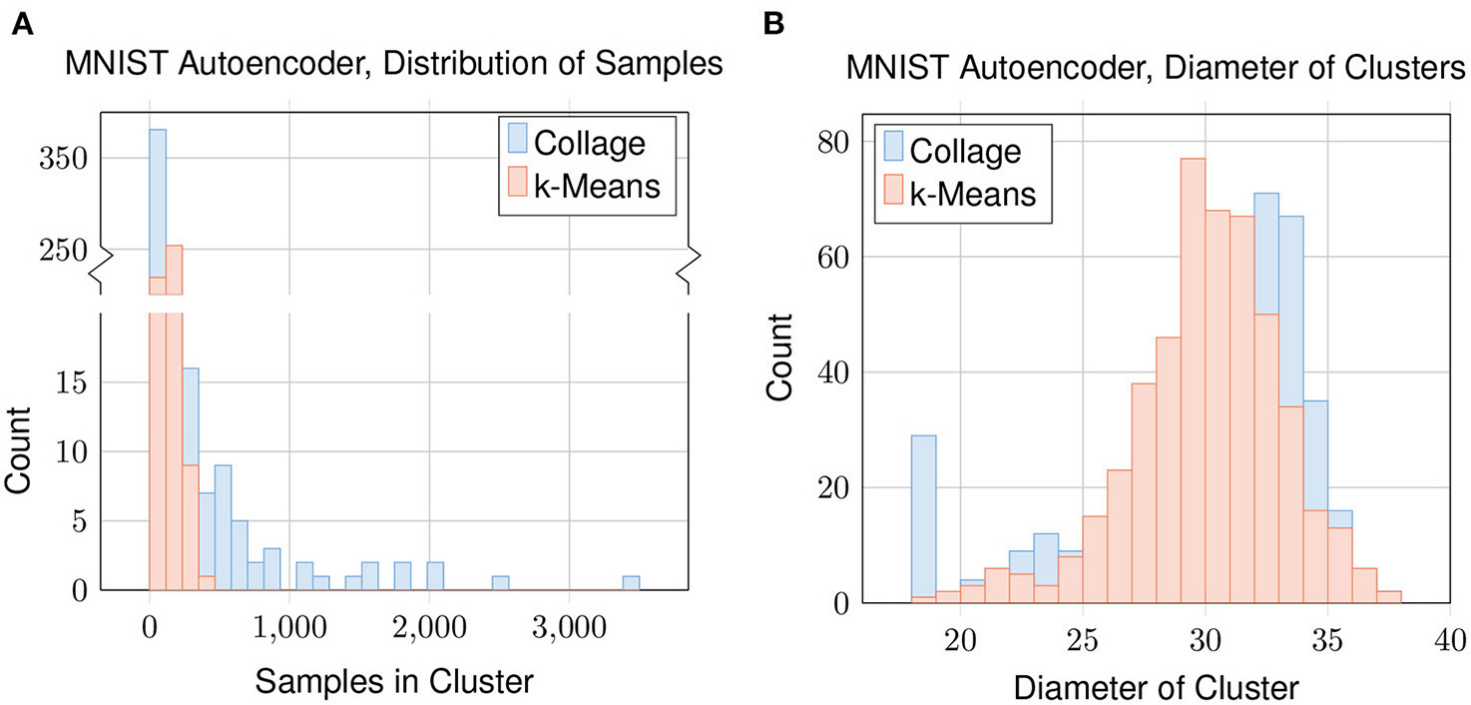
Clustering of the latent encoding of the MNIST dataset: **(A)** distribution of samples in each cluster; and **(B)** distribution of cluster diameters. For Collage, Green Radius = 25. For k-Means, *n*_clusters_ = 483.

To evaluate how the datapoints are being clustered for each algorithm, we plot distributions concerning the samples in each cluster in Figure 9. We look at Collage with green radius = 25 which gives 483 clusters and mean homogeneity of 0.69. We match this by also testing k-Means with *n*_clusters_ = 483. The distribution of the number of samples in each cluster (Figure 9A) shows that Collage has a high concentration of clusters with relatively few samples, and a few clusters containing many samples. The largest cluster contains 3,459 samples, which is enough to represent 6 % of the dataset in one cluster. In contrast, the variance of cluster sizes generated by k-Means is far lower; the number of samples in each cluster being in the low hundreds, with the highest being 353.

Distributions of the cluster diameters (Figure 9B) are similar between Collage and k-Means. An exception is that Collage has a high concentration of clusters with diameter close to or equal to zero. Clusters with zero diameter are singleton clusters.

**Figure 10:**
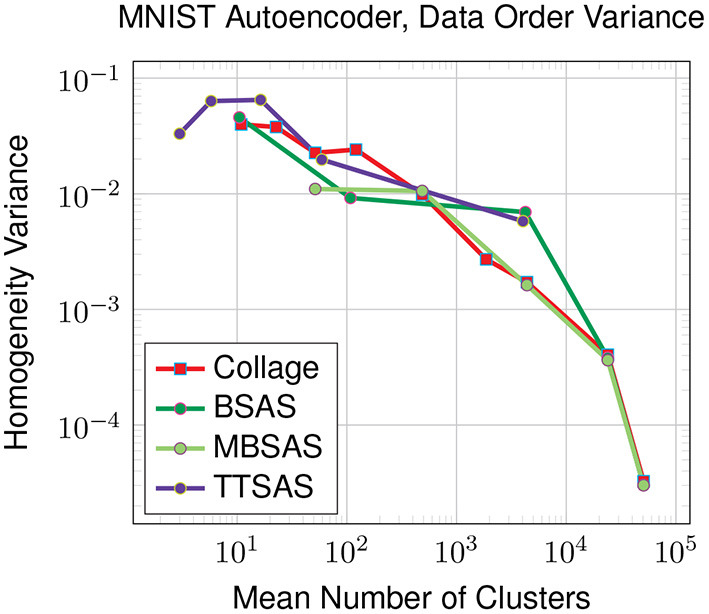
Variance of homogeneity across 10 different data orderings. Latent representations generated for the MNIST dataset by the autoencoder model. For TTSAS, *r*2 is 10 % larger than *r*1.

As the sequential clustering algorithms are susceptible to data ordering, we also investigate the variance in homogeneity over ten different orderings. The results in Figure 10 show that Collage, BSAS and MBSAS are all affected in a similar way, with variances around 0.03 in the worst case for a small number of clusters roughly equalling the number of ground truth classes. While TTSAS claims to provide improved robustness to data ordering compared with BSAS and MBSAS algorithms, we do not see this trend in our experiment. This could be attributed to the highly-diverse nature of our latent representation, although thorough investigation is left for future work.

### 4.6. Augmented MNIST dataset

In this experiment we study how latent representations are affected by augmenting the training dataset. We focus on the MNIST dataset using an autoencoder and augmentations as discussed in Section 3.1. The various losses are plotted in Figure 8B. For the augmented dataset, the training loss is higher than for the original dataset since the augmented dataset has ten times more examples, and is expected to be more difficult to reconstruct as a result of augmentation. However, looking at the validation loss on the augmented dataset, it is much lower than that of the original dataset. Training on the augmented dataset has greatly improved our loss on the validation data, which remains unaugmented: the validation data is the same for both augmented and non-augmented experiments, and is easier to classify than the augmented training data. This explains why the validation loss on the augmented dataset is lower than its training loss.

**Figure 11:**
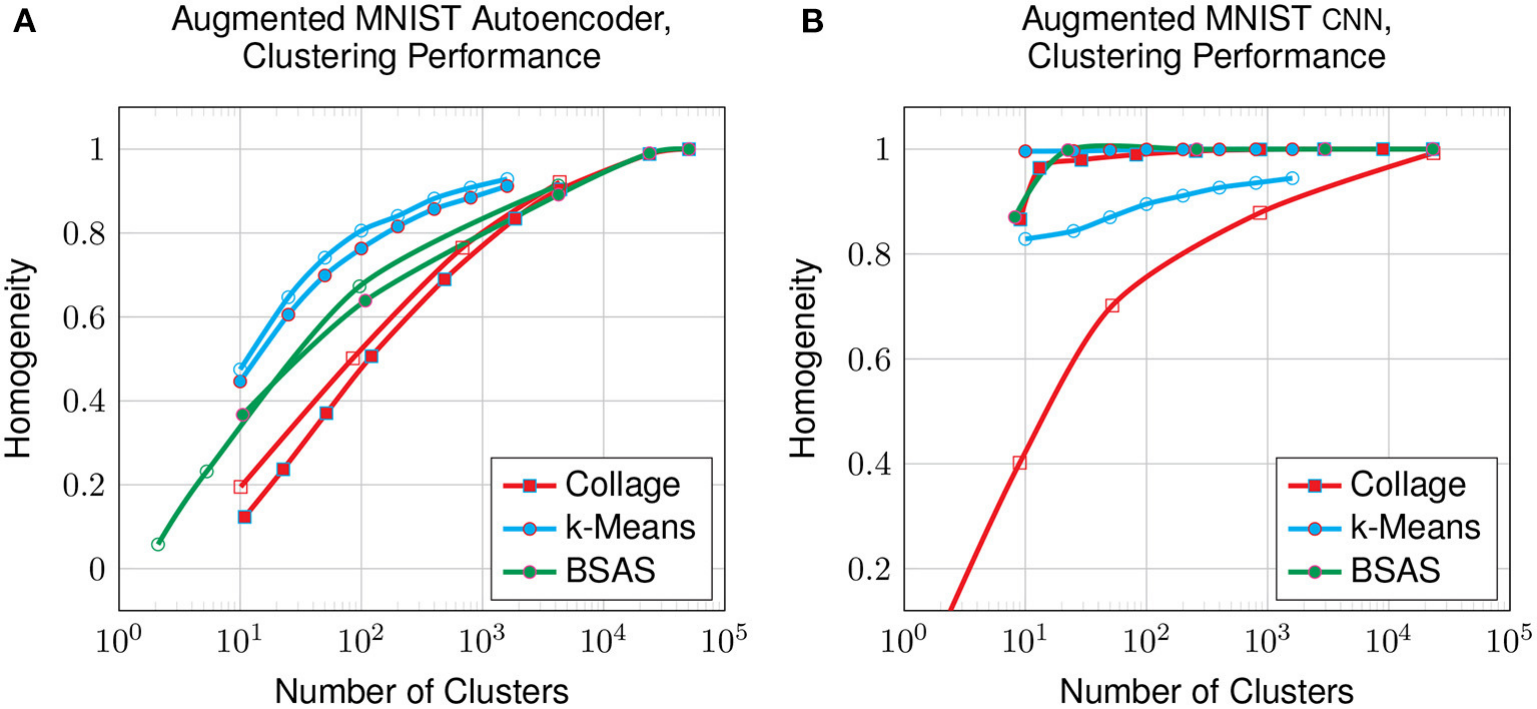
Clustering on the latent-encoded, augmented MNIST dataset: homogeneity compared to the number of clusters on all clustering algorithms from an autoencoder **(A)** and from a CNN **(B)**. Solid datapoints are from the original dataset; hollow datapoints are from the augmented dataset.

Figure 11 shows the clustering performance on the augmented MNIST dataset compared with the original dataset. The data augmentation shows a small improvement to the clustering of the latent space generated by the autoencoder, however, it is hugely detrimental to that generated by the CNN. This suggests that, despite having lower overall performance, the autoencoder's latent representation may be more robust for datasets that have a larger intrinsic spread of datapoints in raw space, which is the effect generated by our augmentation.

## 5. Discussion

Overall, our work shows that clustering in latent spaces depends very heavily on the type of transformation applied on the input space (i.e. loosely speaking the type of neural network used to generate the latent encoding). Whilst CNNs, that aim for generating invariant representations, create conveniently clusterable representations, autoencoders, which aim for equivariance lead to a degradation of clusterability even *vis-a-vis* the original input space. Furthermore, we show that performing clustering online and with a skeletonised number of computations (in our Collage algorithm each new datapoint suffers only as many comparisons as there are cluster centers) comes with a significant degradation to cluster homogeneity in all scenarios. The fact that this occurs even in the CNN case indicates that despite aiming for invariance, datapoints belonging to different classes still lie ‘comparatively close' (with respect to the green and amber radii) in the latent space.

SHADE regularization (Blot, 2020) seeks to minimize *intra*-class invariance, whilst maintaining *inter*-class invariances. This is exactly what is needed to maintain tight clusters of points within the same class, whilst repelling adjacent clusters in the latent space. If this technique is applicable to autoencoders, it could provide a needed boost to the clustering performance of the sensory pipeline.

A possible improvement for the Collage algorithm is to adapt the radii on-the-fly. This presents the challenge of learning to obtain ‘reasonable' clusterings. It is necessary to introduce some metrics to measure this, since we cannot rely on measuring the number of generated clusters, as this would again rely on *a priori* information about the dataset. Finally, merging singleton clusters with their nearest non-singleton clusters could help to reduce the overall number of clusters and increase the number of examples per cluster.

An alternative to calculating the datapoints ownership using L1 geometry is to check each coordinate of a new datapoint against a test cluster center, and then apply pass/fail criteria to determine the ownership to that cluster. For example, if a new datapoint has coordinates (*x*_1_, *y*_1_) and we want to check its ownership against a cluster with center (*x*_2_, *y*_2_), we determine if (*x*_1_−*x*_2_) is greater than a threshold, and if so, we know the datapoint does not belong to the cluster. If the threshold is not exceeded, we proceed to check (*y*_1_−*y*_2_), and only if this is also less than the threshold, we assign the point to the cluster. Geometrically this is equivalent to checking a square, which is simply a 45° rotation of the diamonds we currently use. For the purposes of hardware implementation this is an approach worth considering, however we do not anticipate this to make a significant different to our findings, since the two approaches are near functionally-equivalent.

With the information provided in this work, we believe that a number of important questions arise: Is there a way to obtain both equivariance and better clusterability? Is there an algorithm that can perform the online function of Collage with similar numbers of computations? Is there some compromise between equivariance and invariance that would allow us to train models that both require no prior knowledge of the input space and lead to well-clusterable representations? We hope that this work provides a basis, framework and inspiration for further advances in this area.

## Data availability statement

The raw data supporting the conclusions of this article will be made available by the authors, without undue reservation.

## Author contributions

AW designed and ran experiments, analyzed data, and wrote the manuscript. AS conceived the original pipeline and collage clustering ideas, steered experimental direction, and contributed to the manuscript text. Both authors reviewed and approved the submitted manuscript.
